# Isolation and Characterization of *Mycoplasma mycoides* Subspecies *capri* from Milk of Natural Goat Mastitis Cases

**DOI:** 10.1155/2013/593029

**Published:** 2013-05-20

**Authors:** Vijay Kumar, Rajneesh Rana, Somya Mehra, Pramod Kumar Rout

**Affiliations:** ^1^Central Institute for Research on Goats, Makhdoom, P.O. Farah, Mathura 281122, Uttar Pradesh, India; ^2^Division of Bacteriology and Mycology, Indian Veterinary Research Institute, Izatnagar, Bareilly 243 122, Uttar Pradesh, India

## Abstract

Association of *Mycoplasma mycoides* subspecies *capri* (*Mmc*) with natural goat mastitis has been studied earlier largely by detecting the *Mmc* DNA using molecular methods. However, report on detection of cultivable *Mmc* isolates from natural goat-mastitis milk is still very rare. In this study, *Mmc* was isolated from milk samples (*n* = 171) of goats with or without clinical signs of mastitis. *Mmc* isolates were further characterized by biochemical and species-specific PCR methods. Intra species strain variation was also studied by 16S amplified rDNA restriction analysis (16S ARDRA). The study recovered a total of 6 *Mmc* isolates (3.5%). Three types of intraspecies variants among the recovered *Mmc* isolates were found by 16S ARDRA. The study concluded that *Mmc* may be an etiological agent of mycoplasmal mastitis in Indian goat herds.

## 1. Introduction

*Mycoplasma mycoides* subsp. *capri* (*Mmc*) belongs to the “Mycoplasma mycoides” cluster (*M. capricolum* subsp. *capricolum, M. capricolum* subsp. *capripneumoniae*, *M. mycoides* subsp. *mycoides* large colony type (LC), *M. mycoides* subsp. *mycoides* small colony type (SC), *Mycoplasma* spp. bovine group 7, and *Mmc*) and is reported to cause a pattern of disease (mastitis, arthritis, keratoconjunctivitis, and pleuropneumonia) in goats, similar to those induced by the rest of the species of the mycoides cluster and other mycoplasmas, namely, *M. agalactiae* and *M. putrefaciens* [1, 2]. Mastitis is one of the manifestations of contagious agalactia (CA) [3] and is characterized by clinical signs like heat, pain, swelling, and redness in the udder besides alteration in milk (clot, flakes, discoloration, and reduction or complete cessation of milk yield). CA is prevalent in several regions of the world [4] by causing high morbidity (26.1–100%) in adult goats and 36.5 to 100% in kids [5] along with 25% and 90% mortality in adult goats and kids, respectively [6]. In goat-rearing units the economic loss may reach up to 15–20% [4].

Although *M. agalactiae* is known as the classical etiological agent of CA and/or mastitis, other species of the mycoides cluster have also been found to be associated with goat mastitis in different countries [7–9]. Very recently, Amores et al. [9] have detected *Mmc* using polymerase chain reaction (PCR) from bulk tank milk which was collected from goats exhibiting clinical signs of mastitis from a CA endemic area. However, there is no report about the isolation of *Mmc* from natural goat's mastitis except for the experimental study of Misri et al. [2] and D'Angelo et al. [10]. 

In view of the dearth of information on the association of *Mmc* with goat mastitis on culture bases, the present study was carried out to determine the involvement of *Mmc* as well as intraspecific strain variation by isolation, characterization based on species specific PCR and 16S amplified rDNA restriction analysis (16S ARDRA) pattern.

## 2. Materials and Methods

### 2.1. Classification of Goats for Sampling

Goats exhibiting the clinical signs of mastitis, that is, swollen udder with pain, secreting altered milk, fever, lethargy, and labored breathing, and goats either living in close proximity to goats suffering from mastitis or in herds having a history of CA, were selected for sampling. Goats not exhibiting clinical mastitis were taken as asymptomatic ones and suspected for carriers of mycoplasmas.

### 2.2. Goat Herd, Sample Collection, and Isolation of Mycoplasmas

A total of 171 goats were sampled for milk from five different goat herds of Mathura region (CIRG, Makhdoom; Jhandipur, Chattar Singh Ka Nagla, Keetham and Agra) that facing the problems of CA and/or mastitis. Out of 171 milk samples, 102 were from clinical mastitis and 69 from asymptomatic goats. Milk samples were collected aseptically in sterile vials, that is, before milking affected teats were cleaned with 70% ethanol and then the first 2-3 streams were discarded. 

Isolation of mycoplasmas from milk samples was performed as described by Carmichael et al. [11] with slight modifications. Briefly, milk samples were inoculated into liquid Hank's balanced salt solution (HBSS-L; pH 7.6–7.8) medium and incubated at 37°C under 5% CO_2_ for up to 14 days and subsequently transferred on solid Hank's balanced salt solution (HBSS-S) medium. The probability of L phase variants was ruled out by forward and reversal passages in the artificial medium. 

### 2.3. Biochemical Characterization

A preliminary characterization of the isolates was performed by digitonin sensitivity and growth inhibition tests as per the method described elsewhere [12] and Giemsa method of staining. This was followed by biochemical tests, namely, glucose fermentation, phosphate reduction, gelatin hydrolysis, and film and spot formation test as described earlier [13].

### 2.4. Confirmation of Isolates by PCR

 Genomic DNA of the isolates was extracted from late exponential growth phase using the phenol-chloroform method described by van Kuppeveled et al. [14], and purity and concentration of DNA was checked on 0.7% agarose gel and spectrophotometric analysis according to Sambrook et al. [15].

Mycoplasma isolates were confirmed by employing *Mmc*-specific PCR and the presence of other mycoplasmas was ruled out by conducting the respective species-specific PCRs according to respective protocols described elsewhere as referred in Table 1. The Qiagen PCR core kit was used to perform all the PCRs and consequent PCR products were checked on 2% agarose gel.

### 2.5. Characterization of Intraspecies Strain Variation Using 16S ARDRA

The 16S rDNA of all isolates was amplified by using the universal primer pair pA (5′-AGAGTTTGATCCTGGCTCAG-3′) and pH (5′-AAGGAGGTGATCCAGCCGCA-3′) for 30 cycles (20 sec. 94°C; 15 sec. 57°C; and 30 sec. 72°C) using Qiagen PCR core kit as per Edwards et al. [21]. The resultant amplicon (1500 bp) was purified by using purification kit (Bangalore Genei, India). It was subsequently digested with restriction enzyme *Alu* I (Fermentas, sequence: AG^∧^CT) and the restriction fragments were separated on 3% NuSieve 3 : 1 agar by using the method of Stakenborg et al. [22].

## 3. Results and Discussion

Out of 171 clinical and asymptomatic samples, a total of 45 samples showed fine turbidity and pH shift (acidic) imparting a yellow color to the broth medium within 3 to 10 days indicating the mycoplasma growth. After following the protocol of 4-5 reversal and 3-4 forward passages, the possibility of “L phase variant” was ruled out. Only 6 (3.5%) samples yielded colonies of 1 to 2 mm size exhibiting typical fried egg appearance on HBSS-S medium. Their growth characteristics were indicative of the mycoplasmas. Of six isolates, 5 were recovered from clinical mastitis milk, whereas one (isolate number 6) was from subclinical mastitis milk. These growth evidences were in accordance with Razin and Freundt [23] and Sori et al. [24]. In the study, the isolation rate (3.5%) was found to be in agreement with Ikhloea et al. [25], who obtained similar results of 3.7 to 11%; however, our isolation rate seems to be quite low in contrast to the 25 to 71% obtained by Gil et al. [26].

All the isolates showed purplish-pinkish coccobacillary bodies with pleomorphic shape and size upon Giemsa staining. The isolates passed filtration test through 0.45 *μ*m filter and found to be sensitive to digitonin. Biochemical tests, namely, glucose fermentation and gelatin hydrolysis tests gave positive results, while, film and spot formation test and phosphatase test were negative for all isolates. The isolates exhibited positive growth inhibition test using anti-*Mmc* PG-3 antiserum. On the basis of these results all the isolates were suspected to be of *Mmc*.

**Figure 1:**
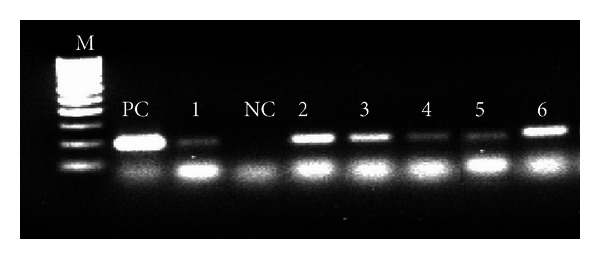
*M. mycoides* subsp. *Capri*-specific PCR exhibiting 195 bp amplicon. M: 100 bp DNA ladder; PC: positive control (*M. mycoides* subsp. *capri* PG-3); NC: negative control; Lanes 1–6: respective isolates.

The PCR amplification in *Mmc*-specific PCR was found positive in all isolates by yielding 195 bp amplicon (Figure 1) the specificity of this PCR was for CAP-21 genomic region [16]. However, none of the species-specific PCR (mentioned in Table 1) except *Mmc* PCR was amplified against any isolate. Thus the presence of any other species (*M. putrefaciens, M. agalactiae, M. capricolum* subsp. *capricolum*, *Mmm* LC, and *M*. bovine group 7) was ruled out, although they are also known to be associated with goat mastitis milk. 

**Figure 2:**
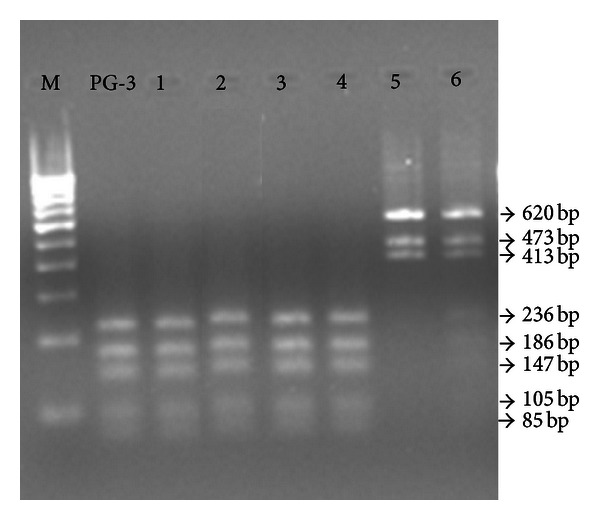
Intra specific strain variation in 16S ARDRA profile among *Mmc* isolates. M: 100 bp DNA ladder; PG-3: *M. mycoides* subsp. *capri* PG-3; Lanes 1–6: respective isolates. The band, that is, 236 bp, 186 bp, 147 bp, and 105 bp are visible very faintly in lane number 6.

*Mmc* isolates were further studied for any intraspecific strain variation using 16S ARDRA. The 16S rDNA upon digestion with *Alu *I exhibited strain variation in *Mmc* isolates by revealing three types of ARDRA patterns (Figure 2). The isolate numbers 1, 2, 3, and 4 showed a similar band pattern as that of *Mmc* PG-3 by yielding 5 bands (236, 186, 147, 105, and 85 bp), while isolate numbers 5 and 6 showed different and unique band patterns by yielding 3 (620, 473, and 413 bp) and 7 (620, 473, 413, 236, 186, 147, and 105 bp) bands, respectively, which were different than the standard strain PG-3. The similarity in band pattern with that of standard strain PG-3 was in agreement with the observations of Stakenborg et al. [22], who observed the same pattern for PG-3 using the same primer and restriction enzyme. However, the different band pattern observed in isolates numbers 5 and 6 was not in agreement with their observation. Our results, that is, different band patterns within species are supported by Monnerat et al. [19] who also found intraspecific strain variation in *lppA *gene of *Mmc* strains by using *Alu* I enzyme. The band pattern different from the reference strain (PG-3) observed by us may be attributed to the presence of different *Alu* I cutting sites in both of the operons (*rrnA* and *rrnB*) as described by Bascunana et al. [27]. 

Although *M. agalactiae* is known as the main causative agent of mastitis [28] along with other species reported earlier, in our case *Mmc* was isolated from goats having clinical mastitis as well as from asymptomatic goats. The isolation of *Mmc* in the present study has also been supported by the detection of *Mmc* from milk collected from clinical mastitis cases in CA endemic area in Spain [9]. Our findings are experimentally supported by Misri et al. [2], who observed the involvement of *Mmc* in development of goat mastitis after following the Koch's postulate. But the present findings are contradictory to earlier reports describing the association of goat mastitis with other mycoplasma species like *M. capricolum* subsp. *capricolum*, *M. putrefaciens*, *M. arginini* and *Mmm* LC [6, 28–31]. Since the present study does not cover a wide geographic area, therefore an isolation work needs to be carried out at a wider level. 

In conclusion, our finding reports the isolation of *Mmc* having intra specific strain variation (in 16S rDNA) from natural mastitis in goats which have not been reported ever and consequently indicates the association and dually favors the earlier report of development of mastitis in goats after the experimental infection of *Mmc*. Also, it reports that, in India, the occurrence of mycoplasmal mastitis in goats may be due to *Mmc* infections as no other mycoplasmal species could be isolated from goat mastitis.

## Figures and Tables

**Table 1:** Details of primers used in study.

Primer	Specificity to species	Sequence	Target gene/region	Annealing temperature	Reference
P4/P6	Mmc* PCR	5′-ACTGAGCAATTCCTCTT-3′ 5′-TTAATAAGTCTCTATATGAAT-3′	CAP-21	46°C, 90 sec	[16]
P4/P5	*Mmm* LC*	5′-ACTGAGCAATTCCTCTT-3′ 5′-TTAAATAAGTTTGTATATGAAT-3′	CAP-21	54°C, 30 sec	[16]
Mag-F/Mag-R	*M*. ag*	5′-CCTTTTAGATTGGGATAGCGGATG-3′ 5′-CCGTCAAGGTAGCGTCATTTCCTAC-3′	16S rRNA	60°C, 60 sec	[17]
MputF/MputR	*M*. put*	5′-AAATTGTTGAAAAATTAGCGCGAC-3′ 5′-CATATCATCAACTAGATTAATAGTAGCACC-3′	*Arc B *	52°C, 15 sec	[18]
MCCPL1-L/MCCPL1-R	Mcc*	5′-AGACCCAAATAAGCCATCCA-3′ 5′-CTTTCACCGCTTGTTGAATG-3′	*LppA *	51°C	[19]
P67BG7-L/P67BG7-R	*Mbg*7*	5′-GGTAATTCGAATAATGATCCT-3′ 5′-TAAGTTTATTGAATTAAAGCG-3′	P67 gene	46°C	[20]

*Mmc: *M*. *mycoides* subsp. *capri*, *Mmm* LC: *M*. *mycoides* susp. *mycoides* large colony type, *M*. *ag*: *M*. *agalactiae*, *Mcc*: *M*. *capricolum* subsp. *capricolum*, *Mbg7*: *M*. bovine group 7.

## References

[1] Cottew GS, Breard A, DaMassa AJ (1987). Taxonomy of the Mycoplasma mycoides cluster. *Israel Journal of Medical Sciences*.

[2] Misri J, Gupta PP, Sood N (1988). Experimental Mycoplasma capri mastitis in goats. *Australian Veterinary Journal*.

[3] Nicholas RAJ (2002). Improvements in the diagnosis and control of diseases of small ruminants caused by mycoplasmas. *Small Ruminant Research*.

[4] Medanet A, Zendulkova D, Pospisil Z (2001). Contagious agalactia of sheep and goats: a review. *Acta Veterinaria Brno*.

[5] De Azevedo EO, De Alcântara MDB, Do Nascimento ER (2006). Contagious agalactia by Mycoplasma agalactiae in small ruminants in Brazil: first report. *Brazilian Journal of Microbiology*.

[6] Saris K, Frey J, Sarris K (1996). Contagious agalactia. *Mycoplasmas of Ruminants: Pathogenicity, Diagnostics, Epidemiology and Molecular Genetics COST 826 Agriculture and Biotechnology*.

[7] Ruhnke HL, Rosendal S, Goltz J, Blackwell TE (1983). Isolation of Mycoplasma mycoides subspecies mycoides from polyarthritis and mastitis of goats in Canada. *The Canadian Veterinary Journal*.

[8] DaMassa AJ, Wakenell PS, Brooks DL (1992). Mycoplasmas of goats and sheep. *Journal of Veterinary Diagnostic Investigation*.

[9] Amores J, Sánchez A, Gómez-Martín A, Corrales JC, Contreras A, de la Fe C (2012). Surveillance of Mycoplasma agalactiae and Mycoplasma mycoides subsp. capri in dairy goat herds. *Small Ruminant Research*.

[10] D’Angelo AR, di Provvido A, di Francesco G (2010). Experimental infection of goats with an unusual strain of Mycoplasma mycoides subsp. Capri isolated in Jordan: comparison of different diagnostic methods. *Veterinaria Italiana*.

[11] Carmichael LE, St George TD, Sullivan ND, Horsfall N (1972). Isolation, propagation, and characterization studies of an ovine Mycoplasma responsible for proliferative interstitial pneumonia. *The Cornell Veterinarian*.

[12] Clyde WA (1964). Mycoplasma spp. Identification based upon growth inhibition by specific antisera. *Journal of Immunology*.

[13] Ernǿ H, Stipkovits L (1973). Bovine Mycoplasmas: cultural and biochemical studies. *Acta Veterinaria Scandinavica*.

[14] Van Kuppeveld FJM, Van der Logt JTM, Angulo AF (1992). Genus- and species-specific identification of mycoplasmas by 16S rRNA amplification. *Applied and Environmental Microbiology*.

[15] Sambrook J, Fritsch EF, Maniatis T (1989). *Molecular Cloning Laboratory Manual*.

[16] Hotzel H, Sachse K, Pfützner H (1996). A PCR scheme for differentiation of organisms belonging to the Mycoplasma mycoides cluster. *Veterinary Microbiology*.

[17] Chávez González YR, Bascuñana CR, Bölske G, Mattsson JG, Molina CF, Johansson KE (1995). In vitro amplification of the 16S rRNA genes from Mycoplasma bovis and Mycoplasma agalactiae by PCR. *Veterinary Microbiology*.

[18] Peyraud A, Woubit S, Poveda JB, De La Fe C, Mercier P, Thiaucourt F (2003). A specific PCR for the detection of Mycoplasma putrefaciens, one of the agents of the contagious agalactia syndrome of goats. *Molecular and Cellular Probes*.

[19] Monnerat MP, Thiaucourt F, Poveda JB, Nicolet J, Frey J (1999). Genetic and serological analysis of lipoprotein LppA in Mycoplasma mycoides subsp. mycoides LC and Mycoplasma mycoides subsp, capri. *Clinical and Diagnostic Laboratory Immunology*.

[20] Frey J, Cheng X, Monnerat MP (1998). Genetic and serological analysis of the immunogenic 67-kDa lipoprotein of Mycoplasma sp. Bovine group 7. *Research in Microbiology*.

[21] Edwars U, Rogall T, Blocker H, Emde M, Bottger EC (1989). Isolation and direct complete nucleotide determination of entire genes. Characterization of a gene coding for 16S ribosomal RNA. *Nucleic Acids Research*.

[22] Stakenborg T, Vicca J, Verhelst R (2005). Evaluation of tRNA gene PCR for identification of mollicutes. *Journal of Clinical Microbiology*.

[23] Razin S, Freundt EA, Krieg NR, Holt JG (1984). The Mollicutes, Mycoplasmatales, and Mycoplasmatacae. *Bergey's Manual of Systematic Bacteriology*.

[24] Sori TDVM, Zeleke ADVM, Gelaye EDVM, Regassa FDVM (2005). Isolation and identification of Mycoplasma mycoides subsp. mycoides small colony type in Eastern Ethiopia. *International Journal of Applied Research in Veterinary Medicine*.

[25] Ikheloa JO, Ajuwape ATP, Adetosoye AI (2004). Biochemical characterization and serological identification of mycoplasmas isolated from pneumonic lungs of goats slaughtered in abattoirs in Northern Nigeria. *Small Ruminant Research*.

[26] Gil MC, De Hermoso Mendoza M, Rey J, Alonso JM, Poveda JB, De Hermoso Mendoza J (1999). Aetiology of caprine contagious agalactia syndrome in Extremadura, Spain. *Veterinary Record*.

[27] Bascunana CR, Mattsson JG, Bolske G, Johansson KE (1994). Characterization of the 16S rRNA genes from Mycoplasma sp. strain F38 and development of an identification system based on PCR. *Journal of Bacteriology*.

[28] Bergonier D, Berthelot X, Poumarat F (1997). Contagious agalactia of small ruminants: current knowledge concerning epidemiology, diagnosis and control. *OIE Revue Scientifique et Technique*.

[29] Bar-Moshe, Rapport E, Bogin E, Lebel E (1982). Vaccination trial against caprine. Mycoplasma mycoids subsp.mycoids (LC type) infection in goats, infectivity trials, vaccination and challenge. *Refuah Veterinarith*.

[30] DaMassa AJ, Brooks DL, Holmberg CA, Moe AI (1987). Caprine mycoplasmosis: an outbreak of mastitis and arthritis requiring the destruction of 700 goats. *Veterinary Record*.

[31] Kumar P, Roy A, Bhanderi BB, Pal BC (2011). Isolation, identification and molecular characterization of Mycoplasma isolates from goats of Gujarat State, India. *Veterinarski Arhiv*.

